# Sorafenib-induced Prostate Volume Reduction, a New Adverse Effect Detected by Imaging: A Pilot Study

**DOI:** 10.5334/jbsr.1607

**Published:** 2018-10-26

**Authors:** Hiroaki Takahashi, Sota Masuoka, Katsuhiro Nasu, Kensaku Mori, Takahiro Kojima, Kuniaki Fukuda, Kazuhiro Takahashi, Toshitaka Ishiguro, Takahiro Hosokawa, Manabu Minami

**Affiliations:** 1 University of Tsukuba, Faculty of Medicine, Department of Diagnostic and Interventional Radiology, Ibaraki, JP; 2 University of Tsukuba, Faculty of Medicine, Department of Urology, Ibaraki, JP; 3 University of Tsukuba, Faculty of Medicine, Department of Gastroenterology, Ibaraki, JP; 4 University of Tsukuba, Faculty of Medicine, Department of Gastroenterological and Hepatobiliary Surgery, Ibaraki, JP; 5 Saitama Children’s Medical Center, Department of Radiology, Saitama, JP

**Keywords:** Sorafenib, Prostate, Adverse effect, Benign Prostatic Hyperplasia

## Abstract

**Background:**

Sorafenib has been used in the treatment of advanced hepatocellular carcinoma (HCC) and renal cell carcinoma (RCC). Sorafenib-associated organ reduction have been reported on imaging, such as thyroid, pancreas and muscle, but there has been no research on prostate volume reduction (PVR).

**Methods:**

We retrospectively analyzed 26 patients (twenty with HCC and six patients with RCC) who underwent sorafenib therapy for 31 to 1225 days (median, 100 days). PVR was estimated by two independent readers using CT volumetry.

**Results:**

The sum of all prostate volumes measured by reader 1 was 24.2 ± 13.8 cm^3^ on the baseline CT and 20.4 ± 10.6 cm^3^ on the follow-up CT (p < 0.001), and that measured by reader 2 was 22.3 ± 13.9 cm^3^ on the baseline CT and 19.2 ± 10.6 cm^3^ on the follow-up CT (p < 0.001). The concordance correlation coefficient for the prostate volume measured by the two readers was 0.95 on the baseline CT scans and 0.94 on the follow-up CT scans. Sorafenib-associated PVR demonstrated slight dependence to the exposure time (r = –0.23). One patient with benign prostatic hyperplasia (BPH) showed PVR (from 80.4 to 61.5 cm^3^ [reader 1]; 83.4 to 61.6 cm^3^ [reader 2]) after sorafenib administration. Sorafenib-associated PVR occurred in patients both with and without underlying liver dysfunction with relative prostate volume changes of 86.7 ± 12.0% and 85.0 ± 9.0%, respectively.

**Conclusion:**

Our study demonstrated significant PVR with sorafenib treatment in patients regardless of the presence of BPH and underlying liver dysfunction.

## Background

Tyrosine kinase inhibitors (TKIs) form an important class of drugs used in molecularly targeted therapy, and they have been widely used in the management of various advanced malignancies [1,2,3]. The therapeutic efficacy of TKIs is achieved mainly by the inhibition of the intracellular kinase domains [1,4]. Sorafenib (Nexavar; Bayer Pharma AG, Leverkusen, Germany) is a small-molecule TKI that inhibits several tyrosine kinases, including vascular endothelial growth factor (VEGF) receptor, platelet-derived growth factor (PDGF) receptor, and the Raf kinases [1,2,3]. Hence, sorafenib has been used to treat advanced hepatocellular carcinoma (HCC), renal cell carcinoma (RCC), and thyroid cancer.

Recently, as the patients’ prognosis with molecularly targeted therapy has been improving, a growing number of unexpected effects are being reported. In addition to the common adverse effects of sorafenib such as diarrhea, hand-foot skin reaction (HFSR), and pancreatitis [2,3,4], some effects that are detected with imaging have also been reported. Thyroid atrophy is one such effect [5]. Skeletal atrophy has also been demonstrated in patients who had received sorafenib for 12 months [6], and several articles have recently reported sorafenib-associated pancreatic atrophy [7,8,9,10,11]. Sunitinib (Sutent; Pfizer, NY, United States), another type of TKI that inhibits the VEGF receptor, has been reported to cause prostate volume reduction (PVR) and thereby reduce obstructive urinary symptoms [12]. It shares similar molecularly targeted effects as sorafenib, and demonstrates some of the same effects, such as thyroid and pancreatic atrophy [11,13,14]. However, there has been no research on whether sorafenib induces PVR, and the correlation between the sorafenib-associated PVR and the other clinical indicators remains unclear. Hence, the purpose of our study was to clarify the effect of sorafenib on prostate volume using a cohort of patients with either HCC or RCC, and to analyze the correlation between PVR and the degree of sorafenib exposure, and the degree of adverse events. Furthermore, we also investigated the correlation between the sorafenib-associated PVR and underlying liver dysfunctions, and benign prostatic hyperplasia (BPH).

## Methods

This retrospective study was approved by our Institutional Review Board (H29-110). The requirement for written informed consent was waived by the Institutional Review Board due to the retrospective nature of the study.

### Study population and data acquisition

Among the male patients with HCC or RCC who underwent computed tomography (CT) scans between April 2008 and March 2017, those who were treated with sorafenib were located. The inclusion criteria for the study were as follows: (i) sorafenib therapy had been continued for at least one month; (ii) a baseline CT scan was obtained before initiating the therapy; and (iii) at least one follow-up CT scan was obtained during sorafenib treatment. The following patients were excluded from the patient group: (i) those who were previously diagnosed with prostate malignancy; and (ii) those who had received hormonal replacement therapy. We identified 26 patients eligible to participate in this study: twenty with HCC and six with RCC.

Clinical data regarding age, underlying liver disease, underlying BPH, the number of days from the beginning of treatment to the follow-up CT scan, the cumulative dose of sorafenib received from the beginning of the treatment to the sequential follow-up CT scans, and the grade of the adverse effects according to the National Cancer Institute Common Toxicity Criteria for Adverse Events version 4.0 were retrospectively collected. Patients were assumed to have underlying BPH if they had been clinically diagnosed with BPH before initiating sorafenib therapy. Patients who had been clinically diagnosed with cirrhosis or other chronic liver dysfunction before initiating sorafenib therapy were considered to have underlying liver disease. The degree of liver fibrosis in patients with liver dysfunction was assessed by the FIB-4 score, which is a noninvasive estimate of liver fibrosis defined as (AST (IU/L) × age (year))/(platelet count (10^9^/L) {\rm{(AST (IU/L)  \times  age (year))/(platelet \ count (1}}{{\rm{0}}^{\rm{9}}}{\rm{/L)  \times  }}\sqrt {ALT(IU\,/\,L)} ) [15,16,17]. Sorafenib was orally administered at 200 mg once daily, 400 mg once daily, 200 mg once, 400 mg once daily, or 400 mg twice daily doses. Dose reduction or interruption of the therapy had been performed according to the grade of the adverse effect. In case of definitive disease progression, sorafenib therapy was permanently continued.

### CT technique and imaging analysis

CT examinations were performed using multi-row detector CT scanners with 256, 64, or 16 detector rows (Brilliance iCT 256, Brilliance 64, Brilliance 16, or M × 8000 IDT 16, Philips Medical Systems, Best, the Netherlands). The collimation width of each detector was 0.625 mm in the iCT256 and Brilliance 64 scanners, and 1.5 mm in the Brilliance 16 and M × 8000 IDT 16 scanners. In all patients, CT scans were performed in the supine position during inspiration breath-hold. The beam pitch was 0.45 to 0.90. Patients with no contraindication for the intravenous injection (IV) of iodinated contrast agents had undergone contrast-enhanced abdominal CT scans. In our study, the contrast-enhanced CT images in the portal venous phase were included for analysis. Patients with renal insufficiency in whom IV contrast agents were contraindicated underwent plain CT scans. The images without contrast enhancement in these patients were also included.

In patients with HCC, the portal venous phase images were automatically initiated 50 seconds after the attenuation of the abdominal aortic blood reached 150 Hounsfields units. In patients with RCC, the portal venous phase images were acquired 80 seconds after the contrast agent was injected. All 20 patients with HCC and five out of six patients with RCC were administered iodinated contrast agents IV for their baseline CT studies. In patients with HCC, 31 follow-up CTs were performed and in patients with RCC 17 follow-up CTs were performed. Of these, 29 and 11 follow-up CTs, respectively, were in the portal venous phase. The remaining CT scans were performed without using an IV contrast agent.

**Figure 1:**
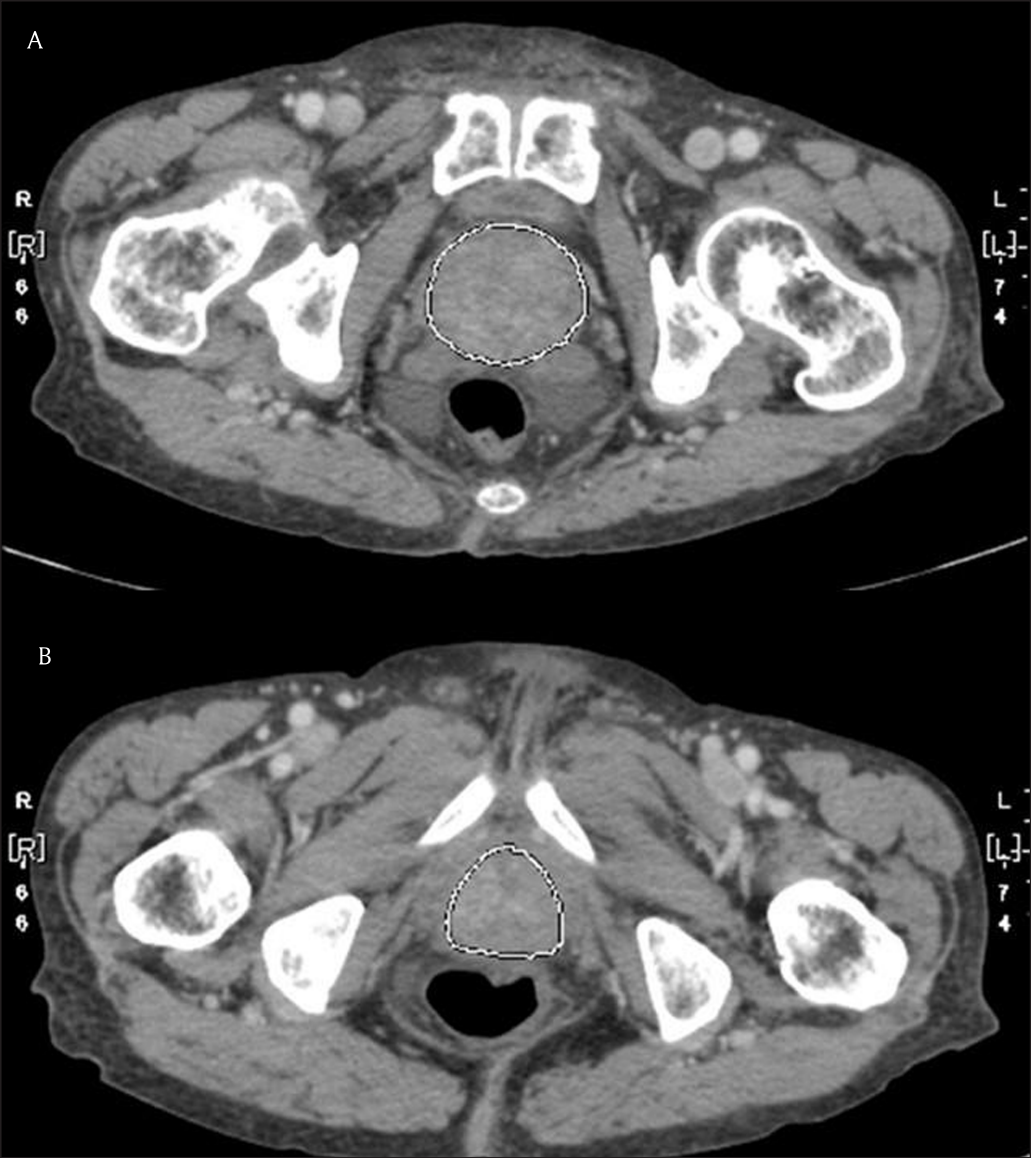
The measurement of the prostate volume using auto-contour measurement.

The Centricity Universal Viewer 6.0 sp7 and Advanced Visualization 3.2 (GE Healthcare, Chicago, IL, USA) were used for image analysis. The volume of the prostate in each CT scan was measured using the “Auto Contour” function, which helped the interpreter to easily perform automated volumetric measurements by area summation. All the 2 mm axial thin slice images of the selected CT scans were anonymized and sent to the workstation. The margins of the prostate were manually outlined by two independent readers (HT, and SM, radiologists with 7 and 4 years of experience, respectively) (Figure 1A, B). The surrounding structures such as vessels and seminal vesicles were carefully excluded from the region of interest (ROI).

### Statistical analysis

The prostate volumes in the baseline and in the final follow-up CT scans that were measured by the two independent readers were assessed by the Wilcoxon signed rank test. The inter-observer agreement for the prostate volume measurements from the baseline and follow-up CT scans were assessed using the concordance correlation coefficient. The agreement between the two readers was visually demonstrated by using Bland-Altman plots with 95% limits. The Wilcoxon rank sum test was used to assess the difference of the prostate volume in the baseline CT and the change in the prostate volume from the baseline CT to the final follow-up CT between patients with liver dysfunction and those without liver dysfunction. The associations between the continuous variables were investigated using the Pearson’s correlation coefficient. In the latter two statistical analyses, we averaged the value of prostate volume measured by the two readers. P values of the respective statistical tests were calculated. All data were analyzed with the statistical software R (version 3.3.2).

## Results

The patient characteristics are summarized in Table 1. Our study included 26 patients for analysis, of whom twenty had HCC and six had RCC. One patient with HCC had been clinically diagnosed with BPH for which he had not undergone any treatment. All the patients with HCC had underlying liver cirrhosis, whereas one of the six patients with RCC had alcoholic hepatitis. Five of the patients with RCC did not have any liver disease.

**Table 1:** Patient characteristics.

	All patients	HCC patients	RCC patients

Total number	26	20	6
Benign prostatic hyperplasia (BPH)	1	1	0
Liver dysfunction^†^	21	20	1
Liver cirrhosis	20	20	0
Viral hepatitis – HBV	4 (19)	4 (20)	0 (0)
Viral hepatitis – HCV	10 (48)	10 (50)	0 (0)
Alcohol	3 (14)	3 (15)	0 (0)
Nonalcoholic steatohepatitis	3 (14)	3 (15)	0 (0)
Chronic liver dysfunction other than cirrhosis	1 (5)	0 (0)	1 (100)
Median age at sorafenib start, years^‡^	71.0 (40–89)	70.0 (40–81)	73.5 (61–89)
Median exposure time to sorafenib, days^‡^	100 (31–1225)	90 (31–1225)	211 (46–674)
Median cumulative sorafenib dose, grams^‡^	47.4 (11.2–364)	46.0 (11.2–364)	50 (16.8–213)
Mean follow-up CT scans^‡^	1.8 (1–7)	1.6 (1–3)	2.8 (1–7)
Diarrhea, grade^†^			
0	15 (58)	10 (50)	5 (83)
1	5 (19)	5 (25)	0 (0)
2	4 (15)	3 (15)	1 (17)
3	2 (8)	2 (10)	0 (0)
HFSR, grade^†^			
0	6 (23)	6 (30)	0 (0)
1	11 (42)	7 (35)	4 (67)
2	4 (15)	3 (15)	1 (17)
3	5 (19)	4 (20)	1 (17)

^†^ Numbers in parenthesis are percentages. ^‡^ Numbers in parenthesis are ranges.

**Figure 2:**
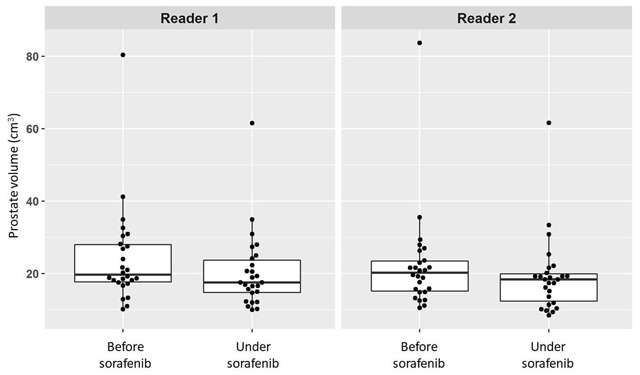
Plotted prostate volumes on the baseline and final follow-up CT as assessed by two readers.

The therapy duration ranged from 31 to 1225 days (median, 100 days) and the cumulative dose of sorafenib ranged from 11.2 to 364 g (median, 47.4 g). The baseline CT scans were performed at a mean of 25.9 days (range 0–148 days) before initiating sorafenib treatment. The prostate volumes before and after sorafenib treatment as measured by the two independent readers are summarized in Table 2 and plotted in Figure 2. The summed prostate volumes of the whole cohort was 24.2 ± 13.8 cm^3^ on the baseline CT, and 20.4 ± 10.6 cm^3^ on the final follow-up CT (p < 0.001) when measured by reader 1; and it was 22.3 ± 13.9 cm^3^ on the baseline CT and 19.2 ± 10.6 cm^3^ on the final follow-up CT (p < 0.001) when measured by reader 2. Significant prostate volume reductions after sorafenib administration were observed by both the readers across the entire cohort, the patients with HCC, and those with RCC.

**Table 2:** Prostate volumes in baseline and final follow-up CT scans as assessed by two independent readers.

	Reader 1				Reader 2			

	Prostate volume in the baseline CT scan (cm^3^)^†^	Prostate volume in the final follow-up CT scan (cm^3^)^†^	P value		Prostate volume in the baseline CT scan (cm^3^)^†^	Prostate volume in the final follow-up CT scan (cm^3^)^†^	P value	

All patients (n = 26)	24.2 ± 13.8 (10.1–80.4)	20.4 ± 10.6 (9.9–61.5)	<0.001	*	22.3 ± 13.9 (10.5–83.7)	19.4 ± 10.6 (8.4–61.6)	<0.001	*
HCC patients (n = 20)	22.9 ± 14.8 (10.1–80.4)	19.3 ± 11.2 (9.9–61.5)	<0.001	*	21.8 ± 15.5 (10.5–83.7)	18.5 ± 11.2 (8.4–61.6)	<0.001	*
RCC patients (n = 6)	28.7 ± 9.4 (17.2–41.2)	24.4 ± 7.9 (12.1–34.9)	0.031	*	24.0 ± 7.3 (15.7–35.5)	21.9 ± 8.3 (11.4–33.4)	0.031	*

^†^ Numbers in parenthesis are ranges. * Statistically significant.

**Figure 3:**
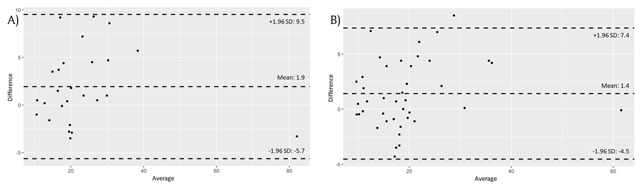
**(A)** Bland-Altman plots of prostate volume as measured by the two readers on baseline CT scans. **(B)** Bland-Altman plots of prostate volume as measured by two readers on the follow-up CT scans.

The concordance correlation coefficient for the prostate volumes measured by the two readers was 0.95 (95% confidence interval [CI]: 0.90, 0.97) on the baseline CT scans and 0.94 (95% CI: 0.89, 0.97) on all the follow-up CT scans. The agreement between the two readers was assessed by the Bland-Altmann plots (Figure 3A, B). The mean difference was 1.9 for the baseline CT scans and 1.4 for all the follow-up CT scans. The 95% limits of agreement between the two readers on the Bland-Altman plots were –5.7 to 9.5 for the baseline CT scans and –4.5 to 7.4 for all the follow-up CT scans.

The relative prostate volume changes on the final follow-up CT scans from the baseline CT scans, obtained by averaging the values measured by the two observers, were 86.4 ± 11.4% (65.1 to 113.1%) in all patients, 85.3 ± 12.2% (65.1 to 113.1%) in HCC patients, and 86.6 ± 9.0% (74.4 to 95.5%) in RCC patients. Of the 26 patients, 23 showed PVR when treated with sorafenib. Three patients showed increased prostate volume of 113.1%, 101.0%, and 103.2%, respectively. The therapy durations and cumulative doses of sorafenib in the three patients were 131 days (52.8 g), 172 days (39.2 g), and 66 days (29.8 g), respectively.

**Figure 4:**
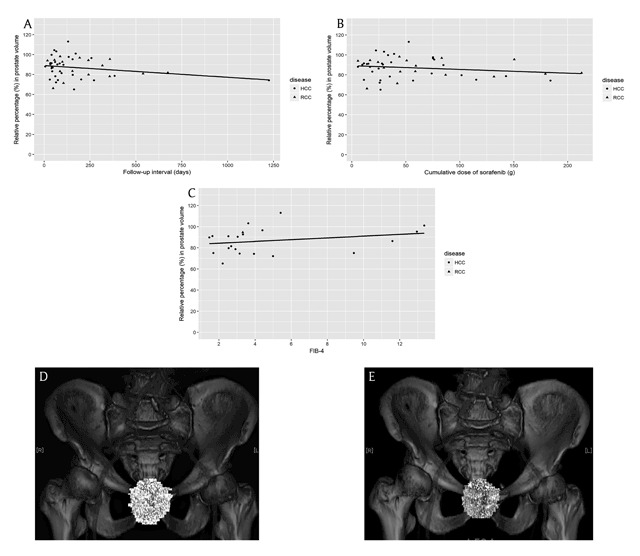
**(A)** The prostate volume change with sorafenib treatment as a function of the exposure time. The correlation coefficient r was –0.23. **(B)** The prostate volume change with sorafenib treatment as a function of the cumulative dose. The correlation coefficient r was –0.18. **(C)** The prostate volume change with sorafenib treatment as a function of FIB-4 score. The analyzed subjects were patients with liver dysfunction (n = 21, twenty patients with HCC and one with RCC). The correlation coefficient r was 0.26. **(D, E)** The prostate volume change after sorafenib treatment in patient with HCC and prostate hyperplasia. The prostate volume reduction after sorafenib administration in this patient was 80.4 cm^3^ (reader 1) and 83.7 cm^3^ (reader 2) on the baseline CT, and was 61.5 cm^3^ (reader 1) and 61.6 cm^3^ (reader 2) in the final follow-up CT. The therapy duration was 31 days; the cumulative dose was 11.2 g.

Each patient’s relative prostate volume change from the baseline CT to the follow-up CT scans was plotted as a function of the follow-up interval (Figure 4A) and as a function of the cumulative dose of sorafenib (Figure 4B). Sorafenib-associated prostate volume change demonstrated dependence on the exposure time (r = –0.23), but not on the cumulative dose (r = –0.18). There was no correlation between the relative volume change of the prostate and the degree of diarrhea (r = –0.12), or the degree of HFSR (r = 0.13).

The prostate volumes of patients with underlying liver dysfunction were compared with those without underlying liver dysfunction. The mean prostate volume on the baseline CT scans was 22.4 ± 14.7 cm^3^ in patients with liver dysfunction (n = 21; twenty patients with HCC patients, and one with RCC), and 26.9 ± 9.1 cm^3^ in patients without liver dysfunction (n = 5; all patients had RCC). The difference between the two groups showed no statistical significance (p = 0.178). The relative prostate volume changes from the baseline CT scans to the final follow-up CT scans were 86.7 ± 12.0% in patients with liver dysfunction and 85.0 ± 9.0% in patients without liver dysfunction (p = 0.753). The FIB-4 score in patients with liver dysfunction ranged from 1.5 to 13.6 (median: 3.3). Sorafenib-associated prostate volume change demonstrated dependence on the FIB-4 score (r = 0.26) in patients with liver dysfunction (Figure 4C).

One patient with HCC also had BPH (patient 1). The prostate volume of this patient was 80.4 cm^3^ (reader 1), and 83.7 cm^3^ (reader 2) on the baseline CT, and 61.5 cm^3^ (reader 1), and 61.6 cm^3^ (reader 2) on the final follow-up CT (Figure 4D, E). The duration of sorafenib therapy was 31 days, and the cumulative dose of sorafenib was 11.2 g. We also investigated from medical records the urinary symptoms of patients in the study cohort. One patient (Patient 2) with HCC had remarked that his urine output had improved after sorafenib therapy. His prostate volume was 24.0 cm^3^ (reader 1) and 23.0 cm^3^ (reader 2) on the baseline CT, and 15.7 cm^3^ (reader 1) and 19.2 cm^3^ (reader 2) on the final follow-up CT. The duration of sorafenib therapy was 1225 days and the cumulative dose was 184 g.

## Discussion

Our study investigated the prostate volume change after sorafenib treatment. Two independent readers measured the prostate volumes of 26 patients who underwent sorafenib therapy (twenty patients with HCC, and six with RCC). Statistically significant PVR was observed in the respective measurements performed by the two readers. The concordance correlation coefficient for the prostate volume calculated by the two readers was substantial on the baseline CT scans (0.95) and the moderate on the follow-up CT scans (0.94), suggesting that the interrater agreement was good.

Our study also revealed that sorafenib-associated prostate volume change tend to be dependent on the exposure time (r = –0.23). The PVR of Patient 1 was observed within a relatively short period of sorafenib exposure (the therapy duration was 31 days). However, three other patients showed increases in their prostate volumes within 180 days of therapy. With long-term sorafenib therapy, the effect of PVR was more certain (Figure 4A, B).

One patient (Patient 1) with HCC who had been clinically diagnosed with BPH also showed PVR after sorafenib administration. He did not take any medication for BPH during the observation period. His prostate volume reduced from 80.4 cm^3^ to 61.5 cm^3^ (reader 1), and from 83.7 cm^3^ to 61.6 cm^3^ (reader 2) after sorafenib therapy (Figure 4D, 4E). On reviewing medical records, we found another patient (Patient 2) with HCC who remarked that his urine output had improved after sorafenib therapy. His prostate volume reduced from 24.0 cm^3^ to 15.7 cm^3^ (reader 1), and from 23.0 cm^3^ to 19.2 cm^3^ (reader 2) after therapy. Hatano et al. demonstrated that RCC patients who had undergone sunitinib administration showed a significant PVR and an improvement in urinary symptoms at week 24 [12]. Therefore, there is a possibility that sorafenib administration may also lead to the amelioration of the urinary symptoms by reducing the prostate volume.

It has been reported that BPH occurred in fewer cirrhotic patients when compared to the general population [18]. Frea et al. reported that the prevalence of BPH in patients with cirrhosis was 41% whereas that in controls was 71% [18]. Mesut et al. demonstrated that the mean prostate volume was 17.9 ± 6.8 cm^3^ in patients with cirrhosis (n = 60) whereas it was 27.6 ± 8.6 cm^3^ in controls (n = 20) [19]. These results could be partly explained by the fact that the prostatic epithelium responds to androgens, the level of which is reduced in patients with cirrhosis [18,19]. This was consistent with the finding in our study that the prostate volume in the baseline CT was larger in patients without liver dysfunction (26.9 ± 9.1 cm^3^) when compared to those with liver dysfunction (22.4 ± 14.7 cm^3^), although the difference between the two groups was not statistically significant (p = 0.178). Our study showed that although sorafenib-associated PVR occurred regardless of the presence of underlying liver dysfunction, it was slightly dependent on the FIB-4 score (r = 0.26). Our findings suggested that PVR with sorafenib therapy could occur even in patients with decreased androgenic activity due to liver dysfunction, but that the degree of PVR might be relatively small in patients with progressive liver fibrosis.

The molecular mechanism by which PVR occurs with sorafenib is considered to be different from that of existing drugs. For instance, Chiu et al. demonstrated that finasteride, a 5α-reductase inhibitor used in BPH, also causes PVR with an average reduction in prostate volume from 39.8 ± 21.1 mL to 33.6 ± 20.5 mL with finasteride treatment when evaluated by ultrasonography [20]. Finasteride-associated PVR is thought to be due to the anti-androgenic effect brought by the reduced formation of dihydrotestosterone from its precursor testosterone [21]. Whereas, sorafenib-associated PVR could be partly explained by the inhibition of the VEGF and PDGF receptors, since previous reports have demonstrated that VEGF and PDGF stimulate the endothelial and epithelial cells in normal prostate glands [12,22,23]. Sorafenib has also been demonstrated to decrease the proliferation of prostate cancer cells by inhibiting the androgen receptor and the Akt signaling pathway, thereby resulting in cell death [24]. It is possible that sorafenib induces this inhibitory function in normal prostate glands as well. The further investigation of these mechanisms of sorafenib that cause PVR could lead to further development of the treatment of BPH that is resistant to therapy.

Our retrospective pilot study had several limitations. First, some CT scans were taken without IV contrast enhancement due to renal dysfunction, and the timings of the portal venous images were different between patients with HCC and those with RCC. This could have caused slight differences in visualizing the boundaries of prostate and might have affected the volume estimated by the two readers to some extent. This was unavoidable since we included RCC patients in the analysis, who were prone to develop renal dysfunction. Second, we did not analyze the correlation between the PVR and the patients’ prognosis. This is because, we considered the prostate volume reduction to be a relatively early onset adverse effect and included patients who were administered sorafenib for shorter durations. Thus, any prognostic information obtained from our study cohort would have had little value. Third, due to the retrospective nature of the study, we could evaluate urinary symptoms in the patients only by investigating their medical records. Further quantitative analysis, including IPSS score, urine flow measurement, and residual urine measurement, would be necessary to assess the correlation between sorafenib-associated PVR and urinary functions.

## Conclusions

Our pilot study has made the novel demonstration of prostate volume reduction with sorafenib therapy. Sorafenib-associated prostate volume reduction could occur in patients regardless of the presence of liver dysfunction or BPH.

## Data Accessibility Statement

The datasets used and/or analyzed during the current study are available from the corresponding author on reasonable request.

## Figures and Tables

**Table:** 

HCC	hepatocellular carcinoma
RCC	renal cell carcinoma
TKI	tyrosine kinase inhibitor
BPH	benign prostatic hyperplasia
HFSR	hand-foot skin reaction
IV	intravenous injection
VEGF	vascular endothelial growth factor
PDGF	platelet-derived growth factor
PVR	prostate volume reduction

## Author Information

This research received the Excellent Exhibition Award at The 54th Autumn Assembly of the Japanese Society of Radiology.
